# (Naphthalen-1-yl){2-[(5,6,7,8-tetrahydronaphthalen-2-yl)carbonyl]phenyl}methanone

**DOI:** 10.1107/S1600536813018035

**Published:** 2013-07-03

**Authors:** J. Kanchanadevi, G. Anbalagan, R. Sivasakthikumaran, A. K. Mohanakrishnan, B. Gunasekaran, V. Manivannan

**Affiliations:** a Department of Physics, Velammal Institute of Technology, Panchetty, Chennai 601 204, India; b Department of Physics, Presidency College (Autonomous), Chennai 600 005, India; c Department of Organic Chemistry, University of Madras, Maraimalai Campus, Chennai 600 025, India; d Department of Physics & Nano Technology, SRM University, SRM Nagar, Kattankulathur, Kancheepuram Dist, Chennai 603 203 Tamil Nadu, India; e Department of Research and Development, PRIST University, Vallam, Thanjavur 613 403, Tamil Nadu, India

## Abstract

The title compound C_28_H_22_O_2_, basically consists of three ring systems, *viz.* a central benzene ring, with a lateral napthalene group to which it subtends a dihedral angle of 66.56 (4)° and a tetra­hydro­pyran ring exhibiting a half-chair conformation. The mol­ecular structure is stabilized by a weak intra­molecular C—H⋯O inter­action, while the crystal packing features weak C—H⋯π contacts.

## Related literature

For the biological activity of diketones, see: Bennett *et al.* (1999 ▶); Sugawara *et al.* (2001 ▶). For related structures, see: Jagadeesan *et al.* (2011 ▶, 2013 ▶)
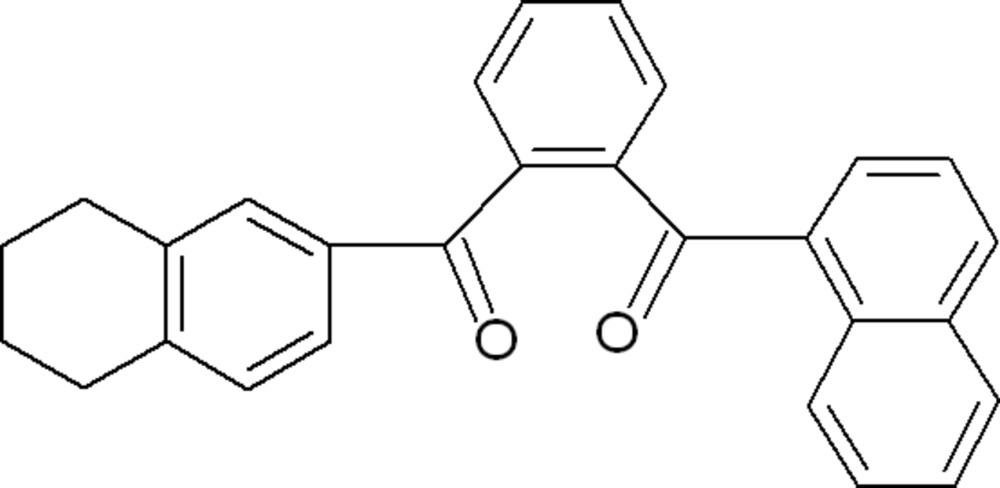


## Experimental

### 

#### Crystal data

C_28_H_22_O_2_
*M* *_r_* = 390.46
Orthorhombic, 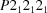

*a* = 10.5625 (5) Å
*b* = 13.6374 (8) Å
*c* = 14.4927 (8) Å
*V* = 2087.6 (2) Å^3^
*Z* = 4
Mo *K*α radiation
μ = 0.08 mm^−1^
*T* = 295 K
0.20 × 0.18 × 0.15 mm

#### Data collection

Bruker APEXII CCD diffractometer
Absorption correction: multi-scan (*SADABS*; Sheldrick, 1996 ▶) *T*_min_ = 0.985, *T*_max_ = 0.989
13047 measured reflections
4998 independent reflections
3511 reflections with *I* > 2σ(*I*)
*R*_int_ = 0.027

#### Refinement

*R*[*F* ^2^ > 2σ(*F* ^2^)] = 0.042
*wR*(*F* ^2^) = 0.100
*S* = 1.03
4998 reflections
272 parameters
H-atom parameters constrained
Δρ_max_ = 0.14 e Å^−3^
Δρ_min_ = −0.13 e Å^−3^

### 

Data collection: *APEX2* (Bruker, 2008 ▶); cell refinement: *SAINT* (Bruker, 2008 ▶); data reduction: *SAINT*; program(s) used to solve structure: *SHELXS97* (Sheldrick, 2008 ▶); program(s) used to refine structure: *SHELXL97* (Sheldrick, 2008 ▶); molecular graphics: *PLATON* (Spek, 2009 ▶); software used to prepare material for publication: *SHELXL97*.

## Supplementary Material

Crystal structure: contains datablock(s) global, I. DOI: 10.1107/S1600536813018035/bg2509sup1.cif

Structure factors: contains datablock(s) I. DOI: 10.1107/S1600536813018035/bg2509Isup2.hkl

Supplementary material file. DOI: 10.1107/S1600536813018035/bg2509Isup3.cml

Additional supplementary materials:  crystallographic information; 3D view; checkCIF report

## Figures and Tables

**Table 1:** Hydrogen-bond geometry (Å, °) *Cg*3 is the centroid of the C12–C17 ring.

*D*—H⋯*A*	*D*—H	H⋯*A*	*D*⋯*A*	*D*—H⋯*A*
C16—H16⋯O1	0.93	2.29	2.856 (2)	118
C4—H4⋯*Cg*3^i^	0.93	2.54	3.419 (1)	159
C25—H25*A*⋯*Cg*3^ii^	0.97	2.94	3.849 (5)	157

Symmetry codes: (i) 
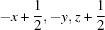
; (ii) 
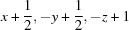
.

**Fig. 1.**
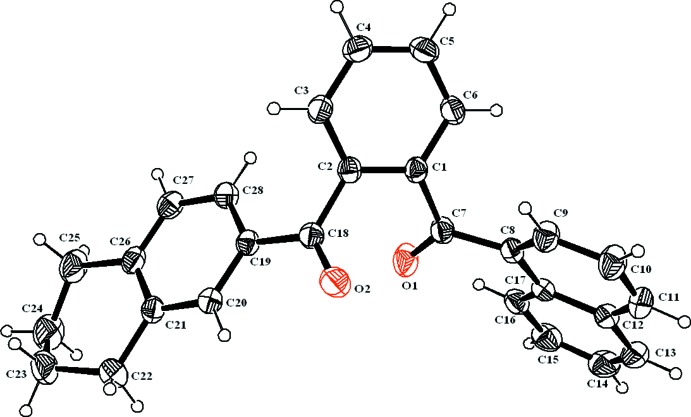
The molecular structure of (I), with atom labels and 30% probability displacement ellipsoids for non-H atoms.

**Fig. 2.**
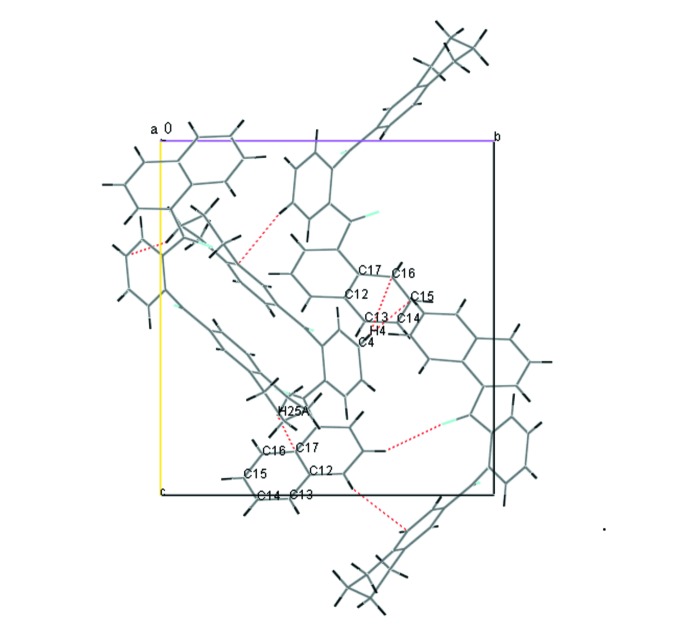
Packing of (I), viewed down the a axis. Hydrogen bonds are shown as dashed lines.

## supplementary crystallographic information

### Comment

In recent days diketones and their derivatives are very important on account of their wide range of applications in biology and medicine. They are known to exhibit antioxidant, antitumour and antibacterial activities (Bennett *et al.*, 1999). Also they are useful as hematopoietic agents in medicine, in particular, in the treatment of cancer, chemotherapy, radiotherapy and drug therapy (Sugawara *et al.*, 2001).

The geometric parameters of the title molecule (Fig. 1) agree well with those of reported similar structures (Jagadeesan *et al.*, 2011, 2013). The central phenyl ring (C1—C6) makes a dihedral angle of 66.56 (4) ° with the napthelene ring (C8—C17) system, while the tetrahydropyran ring exhibits a half-chair conformation.

The molecular structure is stabilized by a weak intramolecular a C—H···O interaction and the crystal packing is controlled by weak C—H···π contacts (Table 1).

### Experimental

To a stirred solution of benzo[*c*]furan (1.0 g, 2.67 mmol) in dry THF (20 ml), lead tetraacetate (LTA) (1.18 g, 2.66 mmol) was added and then stirred at 50 ° C for half an hour. The reaction mixture was then poured into water (200 ml) and extracted with ethyl acetate (2 *x* 20 ml), washed with brine solution and dried (Na2SO4). Removal of solvent in vacuum followed by crystallization from methanol furnished the compound as a colourless solid with a Yield of 94%.

### Refinement

H atoms were positioned geometrically and refined using the riding model with (C—H)_aromatic_ = 0.93 Å and (C—H)_CH2_ 0.97 Å. In all cases *U*_iso_(H) = 1.2Ueq(C.

### Crystal data

**Table:**

C_28_H_22_O_2_	*F*(000) = 824
*M**_r_* = 390.46	*D*_x_ = 1.242 Mg m^−^^3^
Orthorhombic, *P*2_1_2_1_2_1_	Mo *K*α radiation, λ = 0.71073 Å
Hall symbol: P 2ac 2ab	Cell parameters from 5023 reflections
*a* = 10.5625 (5) Å	θ = 2.4–27.9°
*b* = 13.6374 (8) Å	µ = 0.08 mm^−^^1^
*c* = 14.4927 (8) Å	*T* = 295 K
*V* = 2087.6 (2) Å^3^	Block, colourless
*Z* = 4	0.20 × 0.18 × 0.15 mm

### Data collection

**Table:**

Bruker APEXII CCD diffractometer	4998 independent reflections
Radiation source: fine-focus sealed tube	3511 reflections with *I* > 2σ(*I*)
Graphite monochromator	*R*_int_ = 0.027
Detector resolution: 0 pixels mm^-1^	θ_max_ = 28.0°, θ_min_ = 2.4°
ω and φ scans	*h* = −13→8
Absorption correction: multi-scan (*SADABS*; Sheldrick, 1996)	*k* = −17→11
*T*_min_ = 0.985, *T*_max_ = 0.989	*l* = −19→19
13047 measured reflections	

### Refinement

**Table:**

Refinement on *F*^2^	Secondary atom site location: difference Fourier map
Least-squares matrix: full	Hydrogen site location: inferred from neighbouring sites
*R*[*F*^2^ > 2σ(*F*^2^)] = 0.042	H-atom parameters constrained
*wR*(*F*^2^) = 0.100	*w* = 1/[σ^2^(*F*_o_^2^) + (0.0423*P*)^2^ + 0.1006*P*] where *P* = (*F*_o_^2^ + 2*F*_c_^2^)/3
*S* = 1.03	(Δ/σ)_max_ = 0.001
4998 reflections	Δρ_max_ = 0.14 e Å^−^^3^
272 parameters	Δρ_min_ = −0.13 e Å^−^^3^
0 restraints	Extinction correction: *SHELXL97* (Sheldrick, 2008), Fc^*^=kFc[1+0.001xFc^2^λ^3^/sin(2θ)]^-1/4^
Primary atom site location: structure-invariant direct methods	Extinction coefficient: 0.0063 (11)

### Fractional atomic coordinates and isotropic or equivalent isotropic displacement parameters (Å^2^)

**Table:**

	*x*	*y*	*z*	*U*_iso_*/*U*_eq_	
C1	0.33655 (16)	0.99371 (12)	0.82430 (11)	0.0415 (4)	
C2	0.32842 (16)	0.98524 (12)	0.92015 (11)	0.0412 (4)	
C3	0.23477 (17)	1.03566 (13)	0.96667 (13)	0.0490 (4)	
H3	0.2288	1.0302	1.0305	0.059*	
C4	0.15021 (18)	1.09395 (15)	0.91969 (15)	0.0579 (5)	
H4	0.0873	1.1273	0.9519	0.070*	
C5	0.15820 (18)	1.10309 (15)	0.82597 (15)	0.0569 (5)	
H5	0.1011	1.1428	0.7944	0.068*	
C6	0.25110 (17)	1.05336 (13)	0.77827 (13)	0.0517 (5)	
H6	0.2565	1.0599	0.7145	0.062*	
C7	0.42627 (17)	0.92891 (14)	0.77308 (12)	0.0476 (4)	
C8	0.49710 (16)	0.96879 (13)	0.69309 (12)	0.0439 (4)	
C9	0.52520 (18)	1.06701 (14)	0.69107 (14)	0.0543 (5)	
H9	0.4900	1.1078	0.7357	0.065*	
C10	0.6045 (2)	1.10767 (16)	0.62462 (15)	0.0643 (5)	
H10	0.6202	1.1748	0.6243	0.077*	
C11	0.6585 (2)	1.04903 (16)	0.56068 (14)	0.0618 (5)	
H11	0.7140	1.0761	0.5179	0.074*	
C12	0.63253 (16)	0.94787 (15)	0.55742 (12)	0.0498 (5)	
C13	0.68928 (19)	0.88731 (19)	0.49028 (14)	0.0655 (6)	
H13	0.7459	0.9141	0.4481	0.079*	
C14	0.6619 (2)	0.79030 (19)	0.48681 (16)	0.0733 (7)	
H14	0.7003	0.7509	0.4425	0.088*	
C15	0.5770 (2)	0.74932 (16)	0.54904 (15)	0.0669 (6)	
H15	0.5578	0.6829	0.5451	0.080*	
C16	0.52150 (17)	0.80499 (13)	0.61565 (13)	0.0522 (5)	
H16	0.4647	0.7762	0.6565	0.063*	
C17	0.54892 (14)	0.90562 (13)	0.62354 (12)	0.0424 (4)	
C18	0.42905 (17)	0.93173 (14)	0.97279 (12)	0.0446 (4)	
C19	0.39317 (16)	0.85114 (13)	1.03589 (11)	0.0413 (4)	
C20	0.48595 (17)	0.80906 (14)	1.09093 (12)	0.0476 (4)	
H20	0.5684	0.8328	1.0874	0.057*	
C21	0.45937 (17)	0.73290 (14)	1.15083 (12)	0.0504 (5)	
C22	0.5631 (2)	0.6889 (2)	1.20877 (15)	0.0760 (7)	
H22A	0.6168	0.7412	1.2317	0.091*	
H22B	0.6147	0.6468	1.1701	0.091*	
C23	0.5145 (3)	0.62984 (19)	1.28973 (16)	0.0862 (8)	
H23A	0.5833	0.5917	1.3158	0.103*	
H23B	0.4835	0.6740	1.3371	0.103*	
C24	0.4094 (3)	0.5620 (2)	1.26035 (19)	0.0970 (9)	
H24A	0.4404	0.5177	1.2131	0.116*	
H24B	0.3823	0.5229	1.3127	0.116*	
C25	0.2995 (2)	0.61887 (17)	1.22370 (17)	0.0791 (7)	
H25A	0.2545	0.6482	1.2751	0.095*	
H25B	0.2418	0.5739	1.1933	0.095*	
C26	0.33552 (19)	0.69868 (14)	1.15654 (12)	0.0512 (5)	
C27	0.24312 (18)	0.74029 (14)	1.10116 (13)	0.0519 (5)	
H27	0.1605	0.7170	1.1047	0.062*	
C28	0.27079 (17)	0.81506 (13)	1.04115 (12)	0.0468 (4)	
H28	0.2075	0.8415	1.0041	0.056*	
O1	0.44000 (15)	0.84526 (11)	0.79968 (10)	0.0732 (4)	
O2	0.53817 (12)	0.95838 (12)	0.96392 (11)	0.0726 (4)	

### Atomic displacement parameters (Å^2^)

**Table:**

	*U*^11^	*U*^22^	*U*^33^	*U*^12^	*U*^13^	*U*^23^
C1	0.0438 (10)	0.0393 (10)	0.0415 (9)	−0.0032 (8)	0.0041 (7)	0.0052 (8)
C2	0.0430 (9)	0.0389 (10)	0.0416 (9)	−0.0055 (8)	0.0013 (7)	0.0034 (7)
C3	0.0545 (11)	0.0479 (11)	0.0446 (10)	0.0008 (9)	0.0094 (8)	0.0021 (8)
C4	0.0523 (11)	0.0541 (12)	0.0674 (13)	0.0082 (10)	0.0130 (9)	0.0033 (10)
C5	0.0513 (12)	0.0507 (12)	0.0688 (13)	0.0109 (9)	−0.0017 (9)	0.0126 (10)
C6	0.0575 (11)	0.0524 (12)	0.0453 (10)	0.0011 (9)	−0.0028 (9)	0.0104 (9)
C7	0.0552 (11)	0.0437 (11)	0.0438 (10)	0.0001 (9)	0.0037 (8)	0.0060 (8)
C8	0.0478 (10)	0.0430 (10)	0.0409 (9)	0.0035 (8)	0.0012 (7)	0.0055 (8)
C9	0.0651 (12)	0.0444 (11)	0.0535 (11)	−0.0031 (9)	0.0090 (9)	0.0008 (9)
C10	0.0758 (13)	0.0477 (12)	0.0693 (13)	−0.0124 (10)	0.0153 (12)	0.0074 (11)
C11	0.0614 (12)	0.0649 (14)	0.0590 (13)	−0.0073 (11)	0.0136 (10)	0.0149 (10)
C12	0.0450 (10)	0.0607 (13)	0.0436 (10)	0.0060 (9)	0.0009 (7)	0.0061 (9)
C13	0.0587 (12)	0.0831 (18)	0.0547 (12)	0.0140 (11)	0.0094 (10)	0.0021 (11)
C14	0.0760 (15)	0.0790 (18)	0.0649 (14)	0.0293 (13)	0.0060 (11)	−0.0137 (13)
C15	0.0809 (15)	0.0487 (13)	0.0710 (15)	0.0172 (11)	−0.0036 (12)	−0.0058 (11)
C16	0.0599 (11)	0.0448 (11)	0.0519 (11)	0.0078 (9)	−0.0016 (9)	0.0069 (9)
C17	0.0410 (9)	0.0454 (10)	0.0406 (9)	0.0045 (7)	−0.0052 (7)	0.0055 (8)
C18	0.0431 (10)	0.0488 (11)	0.0420 (9)	−0.0021 (8)	0.0002 (7)	0.0033 (8)
C19	0.0457 (9)	0.0416 (10)	0.0367 (8)	−0.0001 (8)	0.0023 (7)	−0.0019 (8)
C20	0.0447 (10)	0.0544 (11)	0.0435 (9)	0.0010 (9)	0.0018 (7)	0.0038 (8)
C21	0.0591 (12)	0.0530 (11)	0.0391 (10)	0.0101 (9)	0.0075 (8)	0.0043 (8)
C22	0.0740 (14)	0.0879 (18)	0.0661 (14)	0.0233 (13)	0.0023 (11)	0.0249 (13)
C23	0.110 (2)	0.0872 (19)	0.0611 (14)	0.0355 (17)	0.0091 (14)	0.0274 (13)
C24	0.121 (2)	0.0741 (18)	0.096 (2)	0.0233 (17)	0.0315 (17)	0.0412 (16)
C25	0.0948 (17)	0.0636 (15)	0.0790 (16)	0.0071 (12)	0.0311 (13)	0.0268 (13)
C26	0.0633 (13)	0.0432 (11)	0.0470 (10)	0.0069 (9)	0.0173 (8)	0.0028 (8)
C27	0.0516 (10)	0.0483 (11)	0.0560 (11)	−0.0068 (9)	0.0096 (9)	−0.0019 (9)
C28	0.0500 (10)	0.0472 (11)	0.0432 (10)	−0.0014 (8)	0.0000 (8)	−0.0009 (8)
O1	0.1049 (12)	0.0478 (8)	0.0669 (9)	0.0165 (8)	0.0289 (8)	0.0166 (7)
O2	0.0455 (8)	0.0879 (11)	0.0844 (10)	−0.0111 (7)	−0.0058 (7)	0.0355 (9)

### Geometric parameters (Å, º)

**Table:**

C1—C6	1.386 (2)	C15—C16	1.361 (3)
C1—C2	1.397 (2)	C15—H15	0.9300
C1—C7	1.493 (2)	C16—C17	1.407 (2)
C2—C3	1.380 (2)	C16—H16	0.9300
C2—C18	1.498 (2)	C18—O2	1.215 (2)
C3—C4	1.376 (3)	C18—C19	1.479 (2)
C3—H3	0.9300	C19—C28	1.385 (2)
C4—C5	1.367 (3)	C19—C20	1.388 (2)
C4—H4	0.9300	C20—C21	1.382 (2)
C5—C6	1.379 (3)	C20—H20	0.9300
C5—H5	0.9300	C21—C26	1.391 (3)
C6—H6	0.9300	C21—C22	1.505 (3)
C7—O1	1.213 (2)	C22—C23	1.513 (3)
C7—C8	1.483 (2)	C22—H22A	0.9700
C8—C9	1.372 (2)	C22—H22B	0.9700
C8—C17	1.435 (2)	C23—C24	1.507 (4)
C9—C10	1.392 (3)	C23—H23A	0.9700
C9—H9	0.9300	C23—H23B	0.9700
C10—C11	1.350 (3)	C24—C25	1.494 (3)
C10—H10	0.9300	C24—H24A	0.9700
C11—C12	1.407 (3)	C24—H24B	0.9700
C11—H11	0.9300	C25—C26	1.509 (3)
C12—C13	1.410 (3)	C25—H25A	0.9700
C12—C17	1.425 (2)	C25—H25B	0.9700
C13—C14	1.355 (3)	C26—C27	1.385 (3)
C13—H13	0.9300	C27—C28	1.372 (2)
C14—C15	1.389 (3)	C27—H27	0.9300
C14—H14	0.9300	C28—H28	0.9300
			
C6—C1—C2	119.15 (17)	C16—C17—C12	117.86 (17)
C6—C1—C7	121.42 (16)	C16—C17—C8	124.35 (16)
C2—C1—C7	118.98 (16)	C12—C17—C8	117.79 (16)
C3—C2—C1	119.25 (16)	O2—C18—C19	122.04 (16)
C3—C2—C18	120.16 (15)	O2—C18—C2	118.26 (16)
C1—C2—C18	120.21 (16)	C19—C18—C2	119.67 (15)
C4—C3—C2	120.75 (17)	C28—C19—C20	118.71 (16)
C4—C3—H3	119.6	C28—C19—C18	122.48 (15)
C2—C3—H3	119.6	C20—C19—C18	118.80 (15)
C5—C4—C3	120.29 (18)	C21—C20—C19	121.89 (16)
C5—C4—H4	119.9	C21—C20—H20	119.1
C3—C4—H4	119.9	C19—C20—H20	119.1
C4—C5—C6	119.83 (18)	C20—C21—C26	118.70 (16)
C4—C5—H5	120.1	C20—C21—C22	120.15 (18)
C6—C5—H5	120.1	C26—C21—C22	121.15 (17)
C5—C6—C1	120.72 (17)	C21—C22—C23	113.45 (19)
C5—C6—H6	119.6	C21—C22—H22A	108.9
C1—C6—H6	119.6	C23—C22—H22A	108.9
O1—C7—C8	122.21 (16)	C21—C22—H22B	108.9
O1—C7—C1	118.32 (15)	C23—C22—H22B	108.9
C8—C7—C1	119.45 (16)	H22A—C22—H22B	107.7
C9—C8—C17	119.25 (16)	C24—C23—C22	111.0 (2)
C9—C8—C7	118.93 (16)	C24—C23—H23A	109.4
C17—C8—C7	121.44 (16)	C22—C23—H23A	109.4
C8—C9—C10	122.27 (19)	C24—C23—H23B	109.4
C8—C9—H9	118.9	C22—C23—H23B	109.4
C10—C9—H9	118.9	H23A—C23—H23B	108.0
C11—C10—C9	119.54 (19)	C25—C24—C23	110.7 (2)
C11—C10—H10	120.2	C25—C24—H24A	109.5
C9—C10—H10	120.2	C23—C24—H24A	109.5
C10—C11—C12	121.43 (18)	C25—C24—H24B	109.5
C10—C11—H11	119.3	C23—C24—H24B	109.5
C12—C11—H11	119.3	H24A—C24—H24B	108.1
C11—C12—C13	120.97 (19)	C24—C25—C26	114.05 (19)
C11—C12—C17	119.64 (18)	C24—C25—H25A	108.7
C13—C12—C17	119.39 (19)	C26—C25—H25A	108.7
C14—C13—C12	120.5 (2)	C24—C25—H25B	108.7
C14—C13—H13	119.8	C26—C25—H25B	108.7
C12—C13—H13	119.8	H25A—C25—H25B	107.6
C13—C14—C15	120.4 (2)	C27—C26—C21	119.38 (17)
C13—C14—H14	119.8	C27—C26—C25	119.43 (18)
C15—C14—H14	119.8	C21—C26—C25	121.17 (18)
C16—C15—C14	120.9 (2)	C28—C27—C26	121.45 (17)
C16—C15—H15	119.5	C28—C27—H27	119.3
C14—C15—H15	119.5	C26—C27—H27	119.3
C15—C16—C17	120.87 (18)	C27—C28—C19	119.86 (17)
C15—C16—H16	119.6	C27—C28—H28	120.1
C17—C16—H16	119.6	C19—C28—H28	120.1
			
C6—C1—C2—C3	0.6 (3)	C11—C12—C17—C8	−1.8 (2)
C7—C1—C2—C3	−171.82 (16)	C13—C12—C17—C8	177.47 (17)
C6—C1—C2—C18	−172.24 (16)	C9—C8—C17—C16	−176.47 (17)
C7—C1—C2—C18	15.3 (3)	C7—C8—C17—C16	10.7 (3)
C1—C2—C3—C4	−0.2 (3)	C9—C8—C17—C12	2.8 (2)
C18—C2—C3—C4	172.73 (17)	C7—C8—C17—C12	−170.07 (16)
C2—C3—C4—C5	−0.3 (3)	C3—C2—C18—O2	−116.9 (2)
C3—C4—C5—C6	0.3 (3)	C1—C2—C18—O2	55.9 (2)
C4—C5—C6—C1	0.2 (3)	C3—C2—C18—C19	61.0 (2)
C2—C1—C6—C5	−0.7 (3)	C1—C2—C18—C19	−126.17 (18)
C7—C1—C6—C5	171.59 (18)	O2—C18—C19—C28	−174.79 (18)
C6—C1—C7—O1	−135.5 (2)	C2—C18—C19—C28	7.4 (2)
C2—C1—C7—O1	36.8 (3)	O2—C18—C19—C20	4.2 (3)
C6—C1—C7—C8	45.7 (3)	C2—C18—C19—C20	−173.60 (16)
C2—C1—C7—C8	−142.05 (17)	C28—C19—C20—C21	−0.3 (3)
O1—C7—C8—C9	−150.0 (2)	C18—C19—C20—C21	−179.40 (16)
C1—C7—C8—C9	28.8 (3)	C19—C20—C21—C26	−0.8 (3)
O1—C7—C8—C17	22.9 (3)	C19—C20—C21—C22	179.53 (18)
C1—C7—C8—C17	−158.31 (16)	C20—C21—C22—C23	162.3 (2)
C17—C8—C9—C10	−1.2 (3)	C26—C21—C22—C23	−17.3 (3)
C7—C8—C9—C10	171.80 (18)	C21—C22—C23—C24	46.4 (3)
C8—C9—C10—C11	−1.4 (3)	C22—C23—C24—C25	−61.6 (3)
C9—C10—C11—C12	2.5 (3)	C23—C24—C25—C26	46.2 (3)
C10—C11—C12—C13	179.9 (2)	C20—C21—C26—C27	1.2 (3)
C10—C11—C12—C17	−0.8 (3)	C22—C21—C26—C27	−179.15 (19)
C11—C12—C13—C14	−178.9 (2)	C20—C21—C26—C25	−177.22 (18)
C17—C12—C13—C14	1.9 (3)	C22—C21—C26—C25	2.4 (3)
C12—C13—C14—C15	0.4 (3)	C24—C25—C26—C27	164.4 (2)
C13—C14—C15—C16	−1.3 (3)	C24—C25—C26—C21	−17.2 (3)
C14—C15—C16—C17	−0.2 (3)	C21—C26—C27—C28	−0.5 (3)
C15—C16—C17—C12	2.4 (3)	C25—C26—C27—C28	177.99 (18)
C15—C16—C17—C8	−178.34 (18)	C26—C27—C28—C19	−0.7 (3)
C11—C12—C17—C16	177.50 (17)	C20—C19—C28—C27	1.1 (3)
C13—C12—C17—C16	−3.2 (2)	C18—C19—C28—C27	−179.88 (16)

### Hydrogen-bond geometry (Å, º)

Cg3 is the centroid of the C12–C17 ring.

**Table:**

*D*—H···*A*	*D*—H	H···*A*	*D*···*A*	*D*—H···*A*
C16—H16···O1	0.93	2.29	2.856 (2)	118
C4—H4···*Cg*3^i^	0.93	2.54	3.419 (1)	159
C25—H25*A*···*Cg*3^ii^	0.97	2.94	3.849 (5)	157

Symmetry codes: (i) −*x*+1/2, −*y*, *z*+1/2; (ii) *x*+1/2, −*y*+1/2, −*z*+1.
